# Two Unconventional Metallothioneins in the Apple Snail *Pomacea bridgesii* Have Lost Their Metal Specificity during Adaptation to Freshwater Habitats

**DOI:** 10.3390/ijms22010095

**Published:** 2020-12-24

**Authors:** Mario García-Risco, Sara Calatayud, Michael Niederwanger, Ricard Albalat, Òscar Palacios, Mercè Capdevila, Reinhard Dallinger

**Affiliations:** 1 Departament de Química, Facultat de Ciències, Universitat Autònoma de Barcelona, E-08193 Cerdanyola del Vallès, Spain; Mario.GarciaRisco@uab.cat (M.G.-R.); oscar.palacios@uab.cat (Ò.P.); merce.capdevila@uab.cat (M.C.); 2 Departament de Genètica, Microbiologia i Estadística and Institut de Recerca de la Biodiversitat (IRBio), Facultat de Biologia, Universitat de Barcelona, Av. Diagonal 643, E-08028 Barcelona, Spain; sarak09@hotmail.com (S.C.); ralbalat@ub.edu (R.A.); 3 Institute of Zoology, Center of Molecular Biosciences, University of Innsbruck, Technikerstraße 25, A-6020 Innsbruck, Austria; michael.niederwanger@uibk.ac.at

**Keywords:** metallothionein, metal-specificity, protein domains, *Pomacea bridgesii*, Mollusca, Gastropoda

## Abstract

Metallothioneins (MTs) are a diverse group of proteins responsible for the control of metal homeostasis and detoxification. To investigate the impact that environmental conditions might have had on the metal-binding abilities of these proteins, we have characterized the MTs from the apple snail *Pomacea bridgesii*, a gastropod species belonging to the class of Caenogastropoda with an amphibious lifestyle facing diverse situations of metal bioavailability. *P. bridgesii* has two structurally divergent MTs, named PbrMT1 and PbrMT2, that are longer than other gastropod MTs due to the presence of extra sequence motifs and metal-binding domains. We have characterized the Zn(II), Cd(II), and Cu(I) binding abilities of these two MTs after their heterologous expression in *E. coli*. Our results have revealed that despite their structural differences, both MTs share an unspecific metal-binding character, and a great ability to cope with elevated amounts of different metal ions. Our analyses have also revealed slight divergences in their metal-binding features: PbrMT1 shows a more pronounced Zn(II)-thionein character than PbrMT2, while the latter has a stronger Cu(I)-thionein character. The characterization of these two unconventional PbrMTs supports the loss of the metal-binding specificity during the evolution of the MTs of the Ampullariid family, and further suggests an evolutionary link of this loss with the adaptation of these gastropod lineages to metal-poor freshwater habitats.

## 1. Introduction

Metallothioneins (MTs) are usually small proteins known for their singularly high content of cysteine residues (Cys), which provide them the capacity for binding essential and non-essential heavy metal ions [1,2,3]. Since MT samples are natively found in association with metals their functions have been related with biological processes involving metal-binding [4]. Some organisms have achieved metal-specific functions of their MTs by harboring multiple forms, each one with a different metal preference [5,6]. Some gastropods, such as *Cantareus aspersus* (also known as *Helix aspersa* or *Cornu aspersum*) and *Helix pomatia*, for instance, exhibit different highly specific MTs devoted to selectively bind Cd(II) (CdMTs) or Cu(I) (CuMTs) [5,7]. Since the expression of CdMTs is upregulated by Cd^2+^ ions, these MTs have been associated with the physiological control of Cd, specifically with Cd detoxification [8,9,10], whereas the expression of CuMTs appears to be activated for controlling Cu homeostasis, which in the case of gastropods is related to the synthesis of the oxygen transporter hemocyanin [5,11].

Interestingly, comparisons of the number and distribution of the Cys motifs (i.e., CxC, CC, and CCC) between MTs have led to conclude that CdMTs and CuMTs of a given group of species derived from duplication events of a common MT ancestor [12], and that changes in the non-coordinating amino acids of the duplicates would have been responsible for the evolution of distinct metal binding preferences [12]. Their frequency and positioning within the sequence, along with their spatial arrangement in the three-dimensional structure determine the metal-selectivity of the protein [7,12,13,14]. In addition to the CdMT and CuMT isoforms, *C. aspersus* and *H. pomatia* express a third MT isoform with intermediate metallic preferences, yielding mixtures of heterometallic metal-complexes when recombinantly synthesized [5,14] or characterized from native MT preparations [15]. These unspecific MTs seem multipurpose proteins capable of maintaining either the physiological levels of metals in the cell [16], transferring them to other metalloproteins [17], or binding toxic heavy metals in order to avoid their deleterious effects [18]. Paradoxically, the amino acid sequences of the three types of gastropod MTs—CdMT, CuMT and unspecific MT—are extremely similar despite their different functionalities. To overcome the difficulties of classifying these clearly different proteins, two criteria were proposed based on MTs’ metal-binding features on the one hand [19,20] and their metal-specific functionality on the other [6,7]. The first criterion is based on metal selectivity of MTs through rendering unique, well-structured, homometallic metal-complexes when binding either divalent (Cd(II) or Zn(II) for Cd/Zn-thioneins) or monovalent metal ions (Cu(I) for Cu-thioneins) [20], and to render a mixture of heterometallic complexes for non-selective MTs somewhere in the middle of this gradual classification [19]. The second criterion is based on physiological evidence indicating a prevalent functionality of the respective MTs in performing metal-specific tasks in favor of the metal ion selectively bound, or in favor of a multi-task performance in case of non-selective MTs [5,7,8,9].

Intriguingly, some gastropod species possess a full set of MTs with different metal binding preferences (CdMTs, CuMTs and unspecific MTs), whereas others like *Megathura crenulata* [21] and *Biomphalaria glabrata* [22] only express unspecific MTs. The diversity of gastropods, as well as of their MTs, is actually so high that the study of gastropod MTs is becoming more and more a model case in the field of MT research [13,23]. The present study adds to this field novel evidence, being devoted to the characterization of two unconventional MTs found in *P. bridgesii* (PbrMT1 and PbrMT2) [13,23]. This species belongs to the group of freshwater apple snails (family Ampullariidae) of the gastropod class of Caenogastropoda, some of them known for their vast ecological impact as pests [24,25]. These snails are also unusual because they possess both a gill and a lung, situated in separated compartments of their mantle cavity. Owing to this particular adaptation, apple snails have assumed an amphibious lifestyle, by which they live predominantly in freshwater swamps and ponds, being also able to temporarily survive under arid conditions buried in the dried-out mud [26].

In a previous work dealing with evolutionary aspects of gastropod MTs including other freshwater snails of the same family (Ampullariidae), PbrMT1 and PbrMT2 were classified as unselective MTs [13]. Based on preliminary analyses and phylogenetic inferences, this work hypothesized that the MTs of freshwater snails might have lost their metal-selective character during adaptation of gastropod families to freshwater habitats with low Cd background concentrations [13]. However, a detailed comparative characterization of the two PbrMTs providing valuable biochemical information about their metal-binding features was missing. To this end, we have thoroughly analyzed the metal-binding abilities of PbrMT1 and PbrMT2 after their recombinant production in metal-supplemented media, including a further experiment in which initially Zn(II)-loaded MT complexes were replaced by Cd(II) to assess their behavior under in vitro conditions. Our approach is based on convincing evidence that heterologous productions of MTs yield metal-MT complexes structurally and functionally equivalent to those isolated from native sources [7,8,14,27,28]. In the present study, all our preparations were characterized by UV-Vis and circular dichroism spectroscopies and mass spectrometry in order to establish the metal-binding features of the *P. bridgesii* MTs, providing experimental evidence to further confirm or reject the inferences made on the basis of our phylogenetic approach. This information will allow us to correlate the binding abilities of these proteins with the requirements of metal handling of *P. bridgesii*, and to speculate how the evolution of unspecific MTs might have provided an adaptive advantage to this species for tolerating variable and fluctuating metal availabilities, improving its adaptation capacity to an amphibious lifestyle.

## 2. Results

### 2.1. Sequence Features of PbrMTs

**Figure 1:**
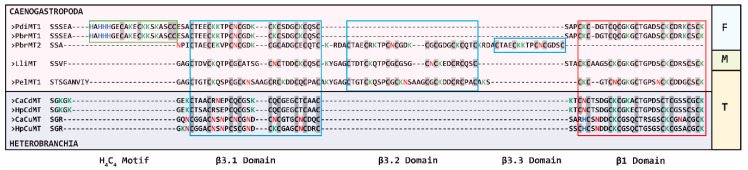
Amino acid alignment of *P. bridgesii* MTs (PbrMT1 and PbrMT2) sequences with other gastropod MTs from species of Caenogastropoda (pink background) and Heterobranchia (purple background) classes (SRA accession number/UniProt accession number): *P. diffusa* PdiMT1 (SRX3488051_2_SRX644696), *L. littorea* LliMT (Q962G0), *P. elegans* PelMT1 (KY646305), *C. aspersus* CaCdMT (A1YZ80; a.k.a. HasMT2) and CaCuMT (A2I9Y4; a.k.a. HasMT1) and *H. pomatia* HpCdMT (P33187; a.k.a. HpoMT2) and HpCuMT (P55947; a.k.a. HpoMT1). β3-domains are boxed in blue, β1-domain in red, and the H_4_C_4_ motif in green. Cysteines are highlighted with a grey background, histidines are colored in blue, lysines in green, and asparagines in red. Colored boxes at the right side of the alignment indicate the habitats of the selected species: in light blue for freshwater (F) habitats, in light green for marine (M) habitats, and in light brown for terrestrial (T) habitats.

As already mentioned above, *P. bridgesii* is a freshwater snail belonging to the gastropod class of Caenogastropoda and specifically, to the apple snail family of Ampullariidae. The amino acid sequences of its two MTs (PbrMT1 and PbrMT2) were aligned with other gastropod MTs (Figure 1). The sequences chosen for the alignment comprise an MT from another freshwater apple snail of the same genus, *Pomacea diffusa* (PdiMT1) [23]; two known Cd-selective MTs from other Caenogastropoda such as the marine periwinkle *Littorina littorea* (LliMT) [29,30], and the round-mouthed snail *Pomatias elegans*, a close relative of *L. littorea*, but adapted to terrestrial conditions [31]. *P. diffusa* is a close relative of *P. bridgesii*, and its PdiMT1 shows a high sequence homology to PbrMT1 and the same domain organization as PbrMT1, although the metal selectivity features of PdiMT1 are still unknown. The MTs from *L. littorea* (LliMT) and *P. elegans* (PelMT1) are Cd-selective and comprise three metal-binding domains, one C-terminal β1 and two N-terminal β3 domains [23]. Additionally, shown in the alignment are two Cd-selective (CaCdMT and HpCdMT) and two Cu-selective MTs (CaCuMT and HpCuMT) from the two terrestrial helicid snails *C. aspersus* and *H. pomatia*, respectively [7,14,32] (Figure 1). These snail species belong to the gastropod class of Heterobranchia which constitutes the sister clade to Caenogastropoda. The alignment in Figure 1 reveals the archetypal structure of gastropod MTs consisting of β3/β1 domains, featuring cysteine motifs like Cx_3_Cx_4_[CxC]x_3–5_[CxC]x_3_[CxC]x_2_C in the N-terminal β3 domain, and [CxC]x_3–4_[CxC]x_3_Cx_5–6_[CxC]x_3_[CxC] in the C-terminal β1 domain [23].

Both PbrMTs show remarkable deviations from the archetypal β3/β1 structure of most other gastropod MTs (Figure 1). PbrMT1 contains additional cysteine (C) and histidine (H) residues in a H_4_C_4_ motif (HxHHHx_2_Cx_3_Cx_6_CC) in its N-terminal region, also shared by PdiMT1 of *P. diffusa* from the same genus, suggesting that the H_4_C_4_ motif may represent a lineage-specific sequence divergence in some MTs of closely related ampullariid freshwater snails. PbrMT2 possesses two tandem duplications (one full-length and one partial) of the β3 domain, but it has lost its β1 domain. Therefore, the domain structure of PbrMT2 is β3.1/β3.2/β3.3 (Figure 1). PbrMT1 and PbrMT2 contain, therefore, an extra number of Cys with respect to the archetypal gastropod MTs, probably conferring to them the capability of binding additional metal ions.

Sequence analysis also revealed that the number of lysine residues (K) is higher in PbrMT1 than in PbrMT2 (14 vs. 9), which in turn contains more asparagine residues (N) compared to PbrMT1 (4 vs. 1). These differences may be significant because previous studies had suggested a connection between the K:N ratio of gastropod MTs and their kind of metal preference [12,22]. Hence, snail MTs with a preponderance of K over N residues would have a binding preference for divalent metals such as Zn(II) and Cd(II), whereas MTs with a predominance of N over K would exhibit a Cu-thionein character [12,19,22].

### 2.2. Production of Metal–PbrMT Complexes

**Figure 2:**
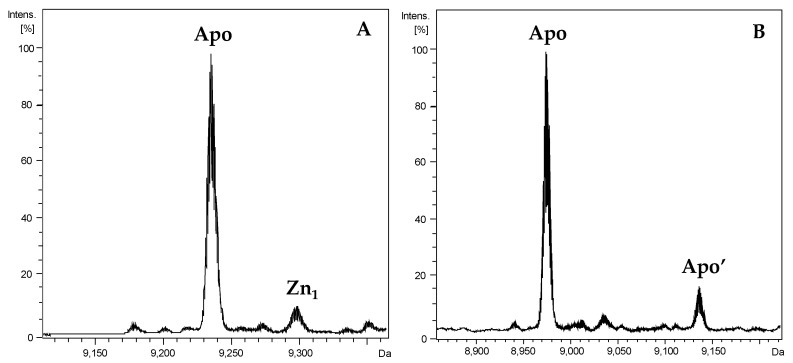
Deconvoluted ESI-MS spectra recorded at acidic conditions (pH 2.4) of (**A**) apo-PbrMT1 and (**B**) apo-PbrMT2 from the Zn-MT productions.

To explore the metal selectivity features of the two *P. bridgesii* MTs, we studied the formation of metal–PbrMT1 and metal–PbrMT2 complexes by the proteins heterologously expressed in *E. coli* and grown in media supplemented with Cu(II), Cd(II) or Zn(II) salts. In order to confirm the identity of the proteins recombinantly produced, apo-PbrMT1 and apo-PbrMT2 forms were obtained from the respective Zn-PbrMT productions under acidic conditions (pH 2.4), and analyzed by ESI-MS. The experimental masses of PbrMT1 and PbrMT2 (9235.5 Da and 8973.3 Da) were coincident with the theoretical masses predicted (9235.51 Da and 8975.20 Da) (Figure 2), ascertaining that the major proportions of the produced proteins corresponded in their masses with the two respective PbrMTs. Here, it should be noticed that recombinant proteins contain two additional amino acids (GS) at their N-term, and therefore the theoretical masses correspond to full length PbrMT1 and PbrMT2 plus those two amino acids. Moreover, in the Zn-supplemented productions, it has to be noted that an additional minor species with an extra mass of 162 Da was detected for PbrMT2. This minor species, named Apo’-PbrMT2 (Figure 2B), did not disappear when acidifying to pH 1, and it was not found neither in PbrMT1 nor in the PbrMT2 productions in media supplemented with Cd(II) or with Cu(II). Preliminary studies suggest that Apo’-PbrMT2 is a minor form artefactually produced only in the PbrMT2 productions with Zn(II) ions, and thereby not affecting the overall characterization of the metal-MTs complexes.

### 2.3. Zn(II) Binding Abilities of the PbrMT Isoforms

**Figure 3:**
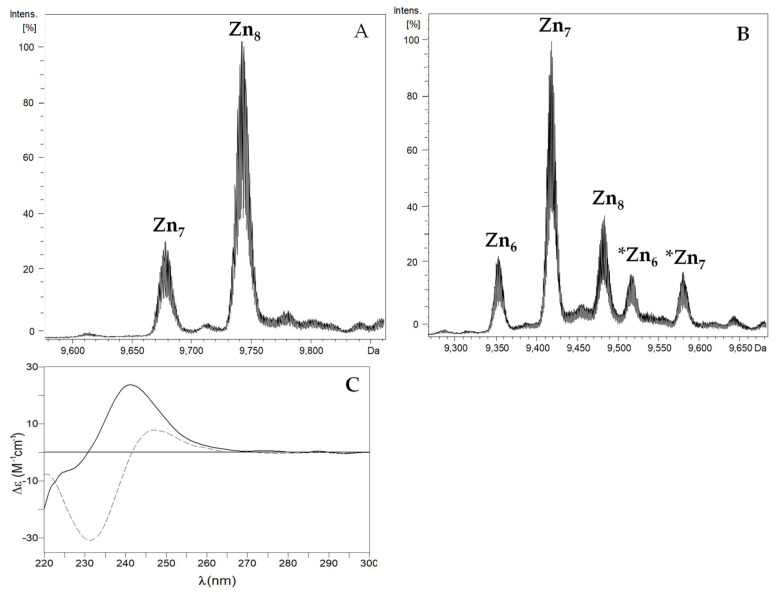
Deconvoluted ESI-MS spectra of the recombinant productions of (**A**) Zn-PbrMT1 and (**B**) Zn-PbrMT2 at neutral pH. (**C**) CD spectra of Zn-PbrMT1 (solid) and Zn-PbrMT2 (dashed) productions. Species with * correspond to metal complexes formed by apo’-PbrMT2.

Both PbrMTs rendered mixtures of several species when synthesized in culture media enriched with ZnCl_2_ (Table 1; Figure 3). In particular, these mixtures consisted of Zn_8_- (major) and Zn_7_- complexes for PbrMT1, and Zn_7_- (major), Zn_8_- and Zn_6_- complexes for PbrMT2. Concordantly, these results match those obtained by ICP-AES (Table 1), and although PbrMT2 has one Cys more than PbrMT1 (23 vs. 22 Cys), both MTs rendered similar metallated species. However, and despite the degree of heterogeneity in speciation of the Zn-PbrMT1, preparation is lower than that of Zn-PbrMT2, the latter shows a higher folding degree with an exciton coupling at ca. 240 nm in the CD spectra in front of the simple Gaussian band at the same wavelength recorded for the Zn-PbrMT1 preparation (Figure 3C).

### 2.4. Cd(II) Binding Abilities of the PbrMT Isoforms

**Figure 4:**
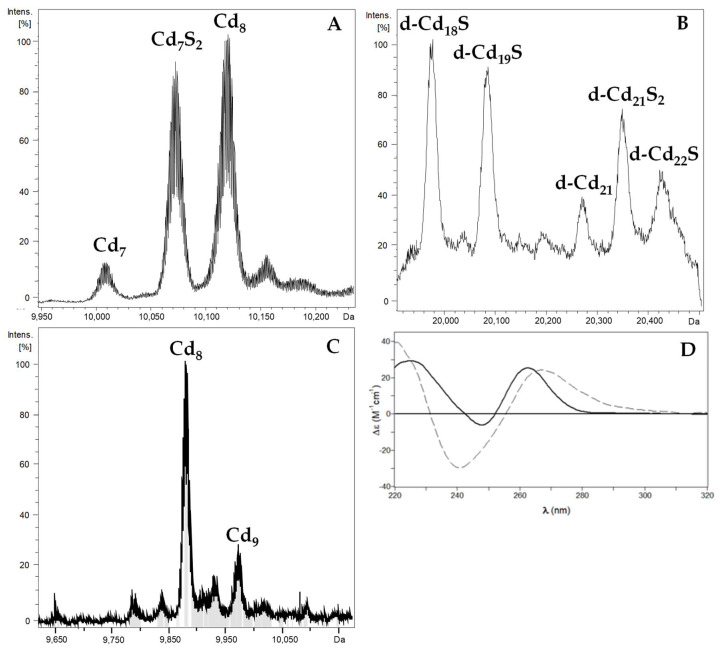
In vivo Cd(II) binding assessment of PbrMT1 and PbrMT2. Deconvoluted ESI-MS spectra of (**A**) Cd-PbrMT1 and (**B**,**C**) Cd-PbrMT2 productions. Dimeric species in the Cd-PbrMT2 production spectrum are marked as “d-“. (**D**) CD spectra of Cd-PbrMT1 (solid) and of Cd-PbrMT2 (dashed) productions.

The recombinant synthesis of PbrMT1 and PbrMT2 in cultures supplemented with CdCl_2_ also rendered a mixture of species (Figure 4), as in the case of the Zn productions. The difference between the conventional ICP values and the acidic ones in both productions (Table 1), as well as the presence of CD bands with maxima at wavelengths higher than 255 nm (Figure 4D) clearly indicate the presence of additional Cd-S^2−^ chromophores [3,33]. On the one side, PbrMT1 rendered Cd_8_- and Cd_7_S_2_- as major species, together with minor Cd_7_-PbrMT1 (Figure 4A). On the other hand, PbrMT2 yielded dimeric species ranging from d-Cd_18_S- to d-Cd_22_S-PbrMT2 (Figure 4B), together with a mixture of monomeric Cd_8_- and Cd_9_-PbrMT2 (Figure 4C) species.

These results already point out that these two MTs are clearly non-Cd-selective and at the same time, barely reveal any differences in their behavior towards Cd(II). To further investigate the Cd(II) binding capabilities of these two MTs, their in vitro Cd(II)-complexes were obtained by adding Cd(II) aliquots to both Zn-PbrMT preparations. As shown in Figure S1 and Table 2, Zn-PbrMT1 shows a gradual non-cooperative replacement of Zn(II) by Cd(II). In terms of CD absorptions, the first 6 Cd(II) equivalents evoked the same effect in the protein folding, leading to the replacement of the initial Gaussian band at ca. 240 nm to a positive CD absorption centered at ca. 265 nm, very similar to the signal recorded for the recombinant Cd-PbrMT1 preparation (Figure S1E). Further additions of Cd(II) only led to small variations in the CD and UV-vis envelopes (Figure S1), suggesting that after 6–7 Cd(II) equivalents added only slight modifications in the structuration of the Cd-PbrMT1 species take place without the incorporation of further Cd(II) ions. The ESI-MS data of this experiment (Figure S2; Table 2) show a subsequent Zn/Cd replacement from the initial preparation, containing Zn_8_- and Zn_7_-PbrMT1 species, to mainly major Zn_1_Cd_7_- and Cd_7_-PbrMT1 after the addition of 7 Cd(II) which remain unaltered in solution for 10 Cd(II) added, without reaching the Cd_8_- major species achieved in the recombinant preparation. 

The Zn/Cd displacement carried out on the Zn-PbrMT2 preparation (Figure S3; Table 3) shows a different pattern from that observed in Zn-PbrMT1, as expected if considering their different amino acid composition (Figure 1) and the speciation of their respective Zn(II) preparations (Figure 3). The addition of Cd(II) to Zn-PbrMT2 provoked a complex progression from an initial CD spectrum showing the characteristic exciton coupling band at ca. 240 nm of certain Zn–MT complexes, to another exciton coupling at ca. 245 nm after 10 Cd(II) equivalents added. This final stage, as expected, is different from that recorded for the recombinant Cd-PbrMT2 sample (Figure S3F) due to the content of labile S^2−^ ions of the latter which contribute to the signal in the 280–300 nm range. UV-vis differences between additions at the latter stages of the experiment showed barely any differences between chromophores, suggesting that the protein did not accept extra metal ions (Figure S3D,E).

It is remarkable that, at after 6 Cd(II) equivalents added, PbrMT2 renders not only the same Zn_1_Cd_7_- species as PbrMT1 but also Cd_8_-PbrMT2. This last species is maintained as one of the important peaks during the whole metal-displacement experiment, as can be seen in the ESI-MS (Table 3; Figure S4). Additionally, unlike PbrMT1, PbrMT2 reaches, at the latter stages, high metallated homonuclear species such as Cd_9_- and Cd_10_-PbrMT2. These results are in agreement with those from the recombinant productions in E. coli and suggest that, although none of the PbrMTs are specifically adapted to capture Cd(II), PbrMT2 can bind more Cd(II) than PbrMT1, in accordance with the former’s higher content in Cys residues. 

### 2.5. Cu(I) Binding Abilities of the PbrMTs

**Figure 5:**
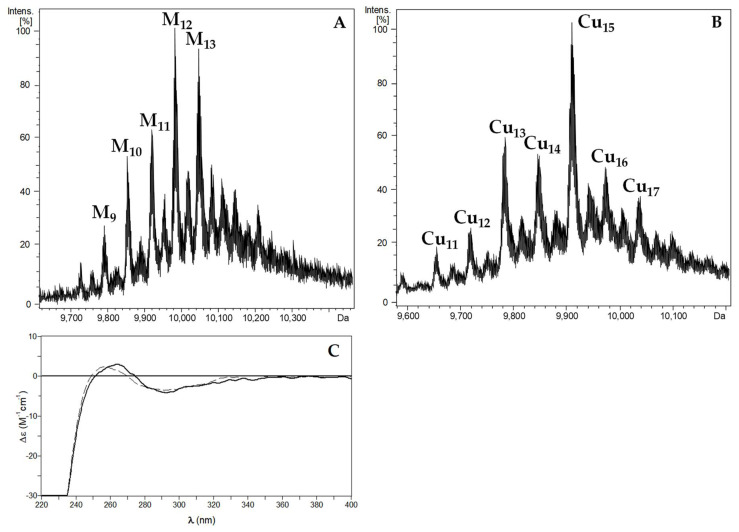
In vivo Cu(I) binding assessment of recombinant PbrMT1 and PbrMT2. Deconvoluted ESI-MS spectra of (**A**) Cu-PbrMT1 and (**B**) Cu-PbrMT2 productions. (**C**) CD spectra of PbrMT1 (solid) and of PbrMT2 (dashed) productions.

The recombinant synthesis of both PbrMT peptides in Cu-supplemented media rendered always a mixture of species (Figure 5), but important differences among both proteins were observed. The ICP-AES results of both Cu-supplemented PbrMT preparations (Table 1) show that, while PbrMT1 renders heterometallic Zn,Cu-species, PbrMT2 yields homometallic Cu(I) species. Moreover, a higher Cu content was detected for PbrMT2 (16.4 Cu), as it was expected if considering that this MT has one more Cys residue than PbrMT1 (12.4 Cu and 2.3 Zn). Consistent with these results, ESI-MS spectra reveal the presence of various metallated species, ranging from M_9_- to M_13_-PbrMT1 (Figure 5A), while for PbrMT2 an intense peak of Cu_15_-PbrMT2 was detected above the minor species ranging from Cu_11_- to Cu_17_-PbrMT2 (Figure 5B). Despite the speciation differences observed, their CD spectra of the two MT preparations are quite similar (Figure 5C), depicting very faint absorption bands that denote a low degree of protein folding about the metal ions.

## 3. Discussion

Sequence analyses reveal unconventional primary structures for both multi-modular MTs of *P. bridgesii*, very divergent from that of the archetypal β_3_/β_1_ MT form of other gastropods (Figure 1). On one hand, PbrMT1 has an additional domain with a H_4_C_4_ motif in its N-terminal region, which has been also found in other gastropod MTs [23], all of them belonging to the freshwater family of Ampullariidae (informal group of Architaenioglossa) form the gastropod clade of Caenogastropoda. It is assumed that a lineage-specific event may have added the H_4_C_4_ motif to the N-terminus of an ancestral Architaenioglossan MT duplicate. This motif increases the number of potential metal-coordinating amino acids (histidines and cysteines), and thereby it could also increase its binding capacity for metal ions. In fact, the recombinant Zn(II)- and Cd(II)-PbrMT productions always rendered mixtures of mainly M_7_- and M_8_-PbrMT species (M = Zn or Cd), which represents up to 33% of increased capacity with respect to the M_6_-MT species rendered by archetypal gastropod β_3_/β_1_ MTs [23]. However, metal binding sites including His residues are known for their preference for Zn over Cd, so this H_4_C_4_ motif may intervene in metal-selectivity features of PbrMT1 [33,34,35]. Overall, we found this peculiar motif extremely interesting, and for that reason, the biochemical properties of this H_4_C_4_ motif in ampullariid MTs and its possible role in modifying metal selectivity features of PbrMT1 will be widely clarified and discussed in a separate study.

On the other hand, structural evolution has led PbrMT2 to lose the C-terminal β1 domain and to tandem-duplicate the β3 domain. These modifications increase the overall number of coordinating cysteines from 18 in the conventional gastropod β_3_/β_1_ MTs [23] to 23, implying a higher number of divalent metal ions that these MTs may be able to bind compared to other gastropod MTs [13]. Our results clearly revealed an increased metal-binding capacity for PbrMT2, ranging from the M_6_-MT species rendered by archetypal gastropod MTs to M_8_-PbrMT2 complexes. Other known mollusk MTs with similarly high Cys contents, such as *Mytilus galloprovincialis* MT-10 (21 Cys) and MT-20 (23 Cys), were recovered binding 7 Cd(II) ions [36,37]. Surprisingly, the presence of 22–23 Cys residues in the *P. bridgesii* metallothioneins provoked the binding of one more M(II) ion, in comparison with the mentioned MTs of *Mytilus*, to render M_8_-species. Moreover, the presence of labile sulfide ligands in the Cd-preparations of both PbrMTs resulted in the absence of a clearly defined M(II)-thionein character (M = Zn, Cd) for these MTs. Interestingly, the analysis of the CD spectra revealed that the Zn-PbrMT2 (major Zn_7_-species, heterogenous speciation) preparation shows an exciton coupling centered at 240 nm which contrasts with the Gaussian band at the same wavelength recorded for the Zn-PbrMT1 (major Zn_8_- and minor Zn_7_-complexes) production. This probably points out to a formation of more compact clusters with 7 Zn(II) ions in PbrMT2 than with 8 Zn(II) ions in PbrMT1. Overall, our results support that the multi-modular MTs of *P. bridgesii* have significantly diverged from the archetypal β_3_/β_1_ gastropod MT variants not only with respect to their primary structure, but also by acquiring a higher metal-binding capacity than the standard gastropod MTs.

Complementary to the data from recombinant productions of metal–MT complexes, the Zn-by-Cd in vitro replacement carried out in both Zn-preparations proceeded similarly. The initial Zn-species led, by a gradual non-cooperative replacement, to final Cd-species with similar CD profiles as those observed in the recombinant Cd-productions. Probably, the absence of the additional S^2−^ ligands in the Zn(II)-preparations observed in the recombinant Cd(II) samples could be the main reason for the recording of different CD signals at the end of both Cd(II) titrations. These Zn/Cd replacement reaction in both proteins revealed that PbrMT1 possesses a higher Zn(II)-thionein character than PbrMT2, and that PbrMT2 exhibits a higher Cd(II) than Zn(II) specificity compared to PbrMT1. While Zn-PbrMT1 productions are reluctant to fully exchange the initial Zn(II), even after the addition of 10 Cd(II) equivalents, Zn-PbrMT2 preparations easily release their Zn(II) metal ions at the early stages of the displacement experiment and render highly metallated homometallic Cd(II)-species at the late stages. With regards to Cu(I) binding, the most remarkable difference between the two PbrMTs has been the significant presence of Zn(II) in the Cu(I)-PbrMT1 production (Table 1), which reinforces the fact that PbrMT1 shows a stronger Zn-thionein character than PbrMT2.

Overall, our analyses have revealed that the two PbrMTs do not exhibit strong metal specificities for any of the tested metals, probably because their overall primary structure and special Cys pattern seem to make them incapable of forming unique homometallic Zn- Cd- or Cu-metallated species. We have therefore experimentally demonstrated that both proteins are unspecific, but with similar although not identical biochemical features. PbrMT1 exhibits a more pronounced Zn(II)-thionein character, while PbrMT2 shows a more explicit Cu(I)-thionein character, which questions the possibility to predict the metal preference of gastropod MTs solely based on sequence features such as the K:N ratios. Our results also address an important aspect in the evolution of metal preferences of gastropod MTs. It has been hypothesized that the ancestral MT of Caenogastropoda might have been Cd-selective [13,23]. Our results imply the loss of the metal-binding specificity during the evolutionary adaptation to freshwater habitats in the lineage of *P. bridgesii* of the Ampullariidae family of Caenogastropoda. It can be argued that due to the low cadmium background levels in the freshwater habitats in which apple snails live [13], the selective pressure for maintaining Cd-selective MTs was no longer effective, leading therefore to the evolution of unspecific MTs. Our results, together with previous studies performed with living snails [38], suggest that the evolution of the two PbrMTs might reflect the needs of *P. bridgesii* to adapt to environmental conditions of metal-poor freshwater habitats, paired with fluctuating availabilities of metal ions due to its amphibious lifestyle. The results in *P. bridgesii* remind some of our previous findings in *B. glabrata*, a freshwater air-breathing snail of the clade of Hygrophila that belongs to the gastropod class of Heterobranchia, the sister class to Caenogastropoda. The MT of *B. glabrata*, too, has lost its Cd selectivity during adaptation of ancestral Hygrophila to freshwater habitats [13,22]. Apparently, this has also happened to PbrMT1 and PbrMT2 of *P. bridgesii*.

## 4. Materials and Methods

### 4.1. Cloning Pomacea bridgesii Metallothioneins for Heterologous Expression

Following an already established methodology in our groups [7,39], cDNAs of PbrMT1 and PbrMT2 (GenBank No. KY963504.1 and KY963505 [13]) were synthesized by Integrated DNA Technologies Company (Coralville, IA, USA). *Bam*HI and *Xho*I restriction sites and 6 or 7 additional 5′-nucleotides were added to both cDNA ends to facilitate the cloning processes. Each synthetic cDNA was PCR-amplified in a 25 µL PCR mixture using Expand High Fidelity PCR system (Roche, Penzberg, Upper Bavaria, Germany) with a common forward primer (5′-TTTTATTGGATCCATGTCTTC-3′ for both *PbrMT1* and *PbrMT2*) and a reverse primer specific for each MT (5′-ATTTTTCTCGAGTCACTTGC-3′ for *PbrMT1*, and 5′-ATTTTTCTCGAGTCAGCAACTG-3′ for *PbrMT2*). PCR conditions for both MT genes were as follows: an initial denaturation step at 94 °C for 5 min was followed by 25 cycles at 94 °C for 30 s, 55 °C for 30 s and 72 °C for 30 s, and a final extension step at 72 °C for 7 min. PCR bands with the expected size were cut and purified with the GenElute™ Gel Extraction Kit (Sigma-Aldrich, St. Louis, MO, USA) following the manufacturer’s instructions. PCR products were digested by *Bam*HI and *Xho*I enzymes, cloned into *Bam*HI-*Xho*I digested pGEX-4T-1 expression vector (GE Healthcare, Chicago, IL, USA) with the DNA Ligation kit 2.1 (Takara Bio Inc., Shimogyo-ku, Kyoto, Japan), and transformed into the *E. coli* DH5α strain.

Plasmid DNA was purified from bacteria cultures using the GeneElute™ Plasmid Miniprep Kit (Sigma-Aldrich), screened for insert presence by digestion with *Sca*I enzyme, and sequenced at the Scientific and Technological Centers of the University of Barcelona, using the Big Dye Terminator v3.1 Cycle Sequencing Kit (Applied Biosystems, Waltham, MA, USA) in an automatic sequencer (ABIPRISM 310, Applied Biosystems). DNA from each recombinant *PbrMT*-pGEX plasmid was used to transform *E. coli* BL21 strain, a protease-deficient strain used for heterologous protein expression.

### 4.2. Production and Purification of Recombinant Metal–PbrMT Complexes

For production of recombinant metal–PbrMT complexes, 500 mL of LB medium with 100 μg/mL ampicillin were inoculated with *E. coli* BL21 cells transformed with the *PbrMT1*-pGEX or *PbrMT2*-pGEX recombinant plasmids. After overnight growth at 37 °C/250 rpm, bacterial cultures were used to inoculate 5 L of fresh LB-100 μg/mL ampicillin medium. MT expression was induced with 100 μM isopropyl-β-D-thiogalactopyranoside (IPTG) for 3 h at 37 °C/250 rpm. After the first 30 min of induction, cultures were supplemented with ZnCl_2_ (300 μM), CdCl_2_ (300 μM), or CuSO_4_ (500 μM) to generate metal-PbrMT complexes. Cells were harvested by centrifugation for 5 min at 9100× *g* (7700 rpm), and bacterial pellets were suspended in 125 mL of ice-cold PBS (1.4 M NaCl, 27 mM KCl, 101 mM Na_2_HPO_4_, 18 mM KH_2_PO_4_) with 0.5% *v/v* β-mercaptoethanol. Suspended cells were sonicated (Sonifier^®^ ultrasonic cell disruptor) 8 min at voltage 6 with pulses of 0.6 s, and then centrifuged for 40 min at 17,200× *g* (12,000 rpm) at 4 °C.

Protein extracts containing GST-PbrMT1 or GST-PbrMT2 fusion proteins were incubated with glutathione sepharose beads (GE Healthcare) for 1 h at room temperature with gentle rotation. After centrifugation at 500× *g* (1553 rpm) for 5 min, pellets of GST-PbrMT fusion proteins bound to the sepharose beads were washed by resuspending them in 30 mL of cold 1xPBS (20 mL for 3 L cultures) bubbled with argon to prevent oxidation. After three washes, GST-MT fusion proteins were digested with thrombin (GE Healthcare, 25 U/L of culture) overnight at 17 °C, enabling separation of the metal–PbrMT complexes from the GST that remained bound to the sepharose matrix. The eluted metal–PbrMT complexes were concentrated with a 3 kDa Centripep Low Concentrator (Amicon, Millipore, MA, USA), and fractionated on a Superdex-75 FPLC column (GE Healthcare) equilibrated with 20 mM Tris-HCl, pH 7.0. Protein-containing fractions were identified by their absorbance at 254 nm, and pooled and stored at −80 °C until use.

### 4.3. Characterization of the Metal–PbrMT1 and Metal–PbrMT2 Complexes

The different metal-MT recombinant preparations were characterized by means of Inductively Coupled Plasma Atomic Emission Spectrometer (ICP-AES), performed in an Optima 4300DV (Perkin-Elmer, Waltham, MA, USA) spectrometer by measuring S at 182.04 nm, Zn at 213.85 nm, Cd at 228.80 nm, and Cu at 324.75 nm. The protein concentration was determined assuming that the total content of S in the proteins comes from both cysteine and methionine amino acids [40]. However, some recombinant productions may render species that include labile S^−2^ anions as a third component on the metal–MT complexes [41]. Therefore, two methodologies were performed to detect these labile sulfide anions. The so-called “conventional ICP” [40] that refers to the standard methodology used to measure the samples employing 1% HNO_3_ solution as a matrix and the “acid ICP” [41] methodology that stands for those samples measured after incubation in 1 M HNO_3_ at 65 °C for 10 min to eliminate labile sulfide anions as H_2_S. Thus, the differences in S content measured via “conventional” and “acid” treatment allowed to detect the presence of labile sulfide anions in the samples. Moreover, the global metal to protein ratios were measured.

The molecular weight of Zn-, Cd- and Cu-MT complexes formed, as well as that of the corresponding apo proteins was determined by Electrospray Ionization Time-of-Flight Mass Spectrometry (ESI-TOF MS) on a Micro TOF-Q instrument (Bruker Daltonics, Bremen, Germany) connected to a Series 1200 HPLC Agilent pump and controlled by the Compass Software. Samples were analyzed under neutral (pH 7.0) and/or acidic (pH 2.4) conditions, using as running buffer a 5:95 mixture of acetonitrile:ammonium acetate (15 mM) and a 5:95 mixture of acetonitrile:formic acid solution, respectively. In acidic conditions, Zn(II) and Cd(II) are released while Cu(I) is kept in the protein complex. Therefore, the characterization of the apo proteins was performed at acidic conditions on the Zn- or Cd-loaded forms. For each analysis, 20 µL of protein solution (of concentrations ranging between 20 and 129 µM) were injected at 30–40 µL·min^−1^ and analyzed under the following conditions: capillary counter-electrode voltage, 3500–5000 V; dry temperature, 90–110 °C; dry gas, 6 L·min^−1^; *m*/*z* range 800–3000. Experimental masses were calculated as previously described [42]. 

Circular Dichroism (CD) measurements were performed in a Jasco spectropolarimeter (Model J-715; Jasco Inc., Easton, MD, USA) interfaced to a computer (J700 software). Electronic absorption was carried out by means of an HP-8453 Diode array UV-Visible spectrophotometer (Hewlett-Packard, Palo Alto, CA, USA). The resultant spectra from both techniques were processed with GRAMS 32 Software (GRAMS/AI v.7.02; Thermo Scientific, Walthman, MA, USA).

### 4.4. In Vitro Metal–Protein Binding Studies

Solutions of the Zn-PbrMT1 (6.06 µM) and Zn-PbrMT2 (6.84 µM) preparations were titrated with Cd(II) at pH 7.0 as previously described [27,43], using a CdCl_2_ standard solution of 1 mM concentration. Essentially, a molar equivalent of metal was added by a stepwise manner to the protein solution. UV-Vis and CD spectra were recorded after each equivalent addition and once the spectrum, and hence the protein folding was stabilized. The procedure continued till metal saturation was reached. All the experiments were performed under strict oxygen-free conditions using argon to saturate the solutions. Aliquots of relevant equivalent additions were taken to execute MS analysis to draw, step by step, the evolution of each MT species throughout the displacement reaction.

## Figures and Tables

**Table 1:** Metal-to-protein ratios found by ICP-AES in the recombinantly produced Zn(II)-, Cd(II)- and Cu(I)-PbrMT1 and PbrMT2 preparations. ESI-MS data were recorded at neutral pH and are shown in several figures along this work.

MT	Supplemented Metal	M/Protein ^a^ (Conventional)	M/Protein ^b^ (Acidic)	ESI-MS Results ^c,d^
PbrMT1 (22 Cys)	Zn	7.4 Zn	n.m.	Zn_8_-(major) > Zn_7_-
	Cd	5.3 Cd	8.6 Cd	Cd_8_- ~ Cd_7_S_2_-(major) > Cd_7_-
	Cu	2.3 Zn;12.4 Cu	n.m.	M_8_- to M_13_-
PbrMT2 (23 Cys)	Zn	6.4 Zn	7.1 Zn	Zn_7_-(major) > Zn_8_- > Zn_6_- > Zn_6_-* > Zn_7_ *
	Cd	6.9 Cd	9.0 Cd	d-Cd_18_S- to d-Cd_22_S- Cd_8_-(major) > Cd_9_-
	Cu	16.4 Cu	n.m.	Cu_9_- to Cu_17_-

^a^ Metal-to-protein ratios obtained from conventional ICP. Zn, Cd and Cu were measured in all cases but only those results different than zero are shown. ^b^ Metal-to-protein ratio obtained from acidic ICP. Zn, Cd, and Cu were measured in all cases but only those results different than zero are shown. n.m. stands for “not measured”. ^c^ d stands for dimeric species. ^d^ species with * correspond to metal complexes formed by apo’-PbrMT2.

**Table 2:** Species detected by ESI-MS during the addition of Cd(II) to the Zn-PbrMT1 preparation at pH 7.0. The corresponding ESI-MS spectra are shown in Figure S2.

Zn + Cd		Cd(II) Equivalents					
		0	1	3	5	7	10
7	Zn_7_-PbrMT1	●	✖				
8	Zn_8_-PbrMT1	✔	●				
8	Zn_7_Cd_1_-PbrMT1		✔				
7	Zn_6_Cd_1_-PbrMT1		●				
8	Zn_6_Cd_2_-PbrMT1		●	✖			
8	Zn_5_Cd_3_-PbrMT1		✖	✔			
8	Zn_4_Cd_4_-PbrMT1			✔			
8	Zn_3_Cd_5_-PbrMT1			●	✔		
8	Zn_2_Cd_6_-PbrMT1			✖	✔		
7	Zn_1_Cd_6_-PbrMT1				●	✖	✖
8	Zn_1_Cd_7_-PbrMT1				●	✔	✔
7	Cd_7_-PbrMT1					✖	●

✔ denotes major species, ● denotes intermediate species, ✖ denotes minor species. The terms “major”, “intermediate” and “minor” species refer to their signal intensity in the corresponding MS spectra.

**Table 3:** Species detected by ESI-MS during the addition of Cd(II) to the Zn-PbrMT2 preparation at pH 7.0. The corresponding ESI-MS spectra are shown in Figure S4.

Zn + Cd		Cd(II) Equivalents					
		0	2	4	6	8	10
7	Zn_7_-PbrMT2	✔					
8	Zn_8_-PbrMT2	●					
8	Zn_6_*-PbrMT2	●					
9	Zn_7_*-PbrMT2	✖	●				
8	Zn_5_Cd_3_-PbrMT2		✔				
7	Zn_4_Cd_3_-PbrMT2		●				
8	Zn_4_Cd_4_-PbrMT2		●	●			
8	Zn_3_Cd_5_-PbrMT2		✖	✔	✖		
8	Zn_2_Cd_6_-PbrMT2			●	●		
8	Zn_1_Cd_7_-PbrMT2			●	✔	●	✖
9	Zn_1_Cd_8_-PbrMT2				●	●	●
8	Cd_8_-PbrMT2				✔	✔	✔
10	Zn_1_Cd_9_-PbrMT2					✖	●
9	Cd_9_-PbrMT2				✖	✖	●
10	Cd_10_-PbrMT2						●

✔ denotes major species, ● denotes intermediate species, ✖ denotes minor species. The terms “major”, “intermediate” and “minor” species refer to their signal intensity in the corresponding MS spectra.

## Data Availability

Additional data for this work can be found in the Supplementary material. For access to raw data, please contact the corresponding author.

## Supplementary Materials

The following are available online at https://www.mdpi.com/1422-0067/22/1/95/s1.
