# Supplementary material for: Two Unconventional Metallothioneins in the Apple Snail Pomacea bridgesii Have Lost Their Metal Specificity during Adaptation to Freshwater Habitats
Source: Int J Mol Sci. 2020 Dec 24;22(1):95. doi: 10.3390/ijms22010095 (PMC7796288; doi:10.3390/ijms22010095)
Supplement: Supplementary file 1 [file ijms-22-00095-s001.pdf]

# SUPPLEMENTARY MATERIAL

## Two unconventional metallothioneins in the apple snail *Pomacea bridgesii* have lost their metal specificity during adaptation to freshwater habitats

Mario García-Risco[a], Sara Calatayud[b], Michael Niedwerwanger[c], Ricard Albalat[b], Òscar Palacios[a], Mercè Capdevila[a] and Reinhard Dallinger[c,*]

[a] *Departament de Química, Facultat de Ciències, Universitat Autònoma de Barcelona, E-08193 Cerdanyola del Vallès, Spain.*
[b] *Departament de Genètica, Microbiologia i Estadística and Institut de Recerca de la Biodiversitat (IRBio), Facultat de Biologia, Universitat de Barcelona, Av. Diagonal 643, E-08028, Barcelona, Catalonia, Spain.*
[c] *Institute of Zoology and Center of Molecular Biosciences, University of Innsbruck, Technikerstraße 25, A-6020 Innsbruck, Austria.*

Corresponding author email: merce.capdevila@uab.cat

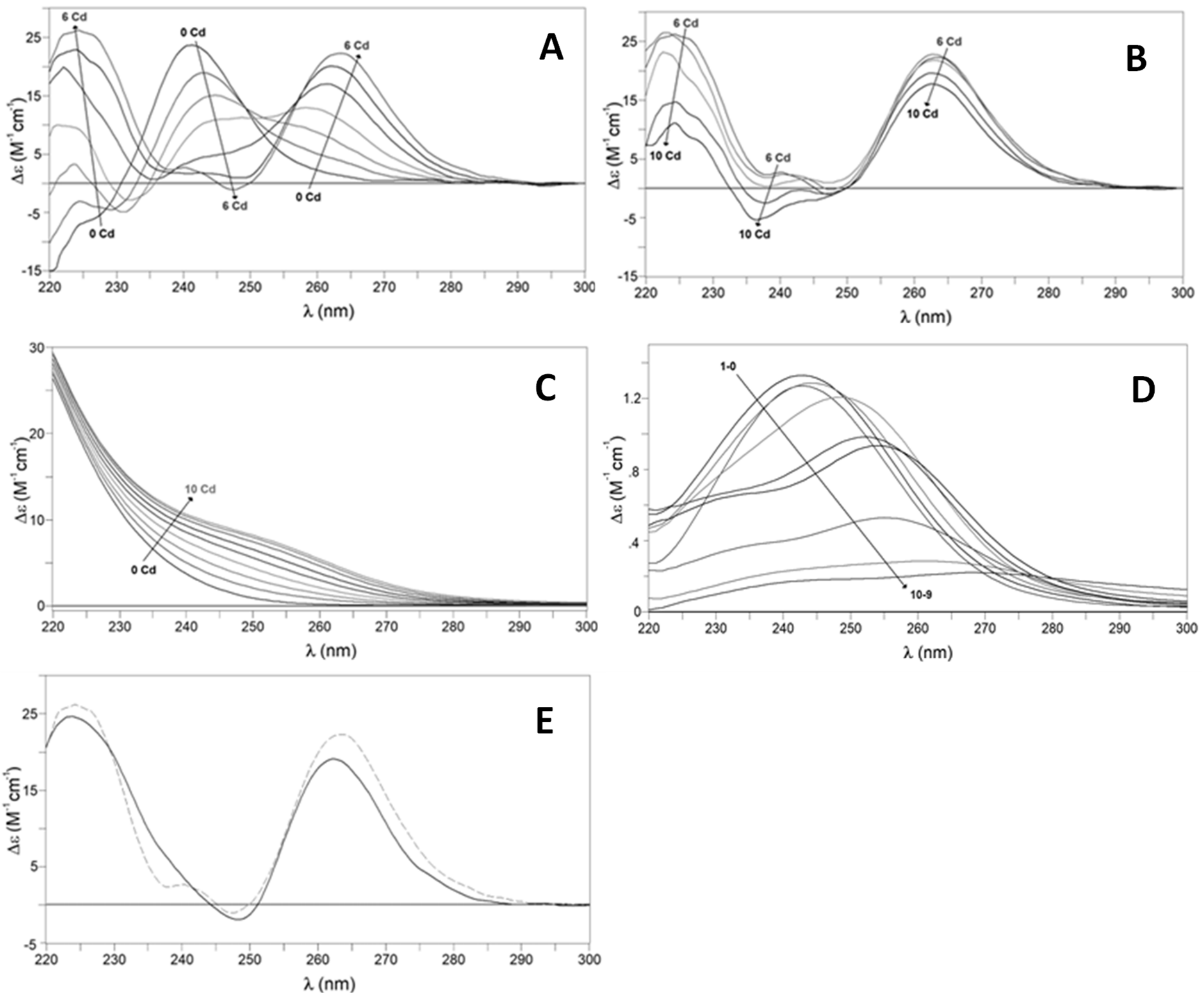


**Figure S1**. Spectroscopic characterization of the Zn/Cd replacement in the Zn-PbrMT1 preparation followed by (A and B) CD and (C) UV-vis. (D) UV-Vis difference spectra. (E) Comparison of CD spectra of *in vivo* Cd-PbrMT1 preparation (solid line) and the *in vitro* Cd-PbrMT1 spectrum measured after adding 7 equivalents of Cd(II) to the Zn-PbrMT1 preparation (dashed line)

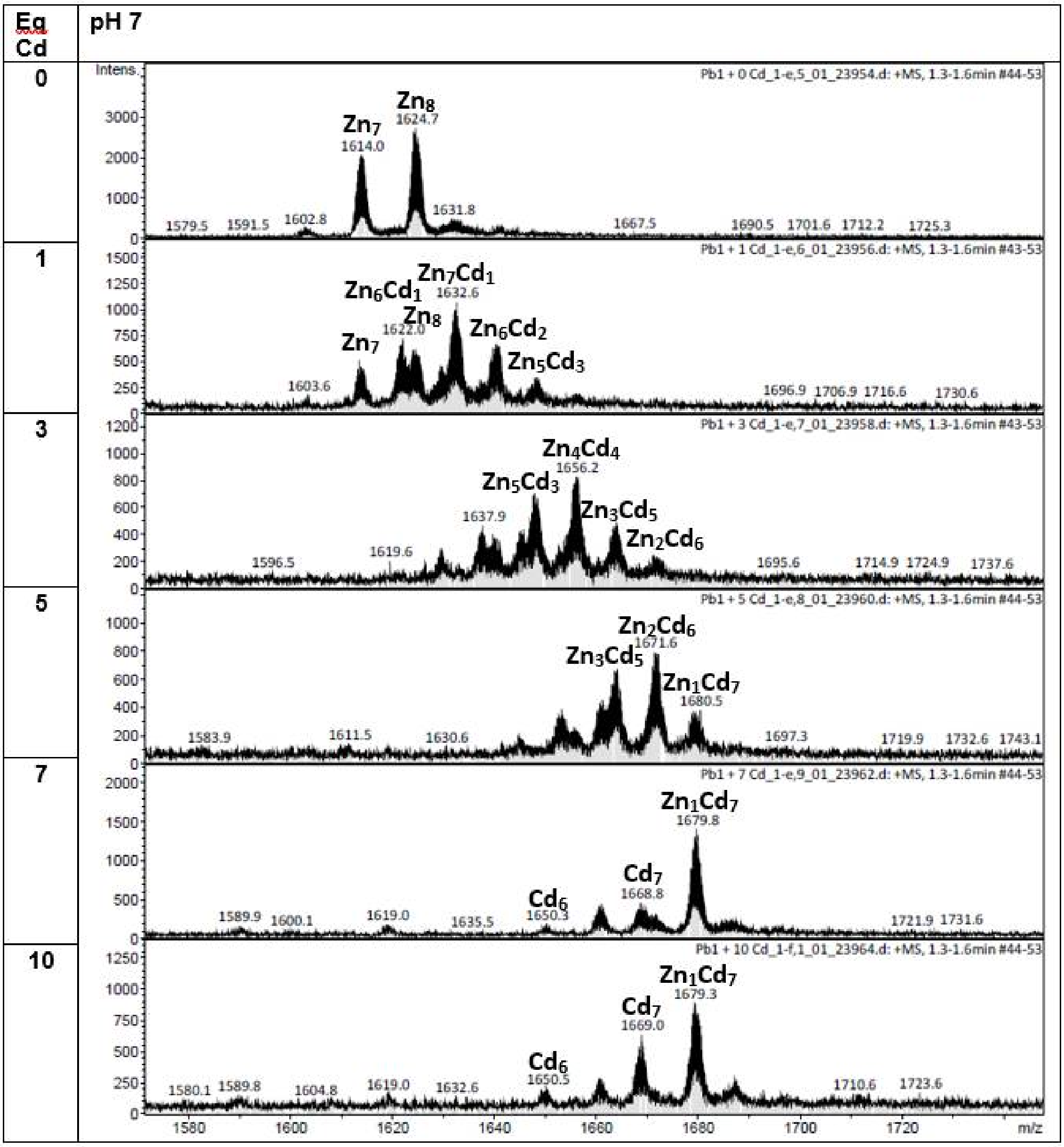


**Figure S2**. ESI-MS spectra of the Zn/Cd replacement experiment performed on the Zn-PbrMT1 production at pH 7.

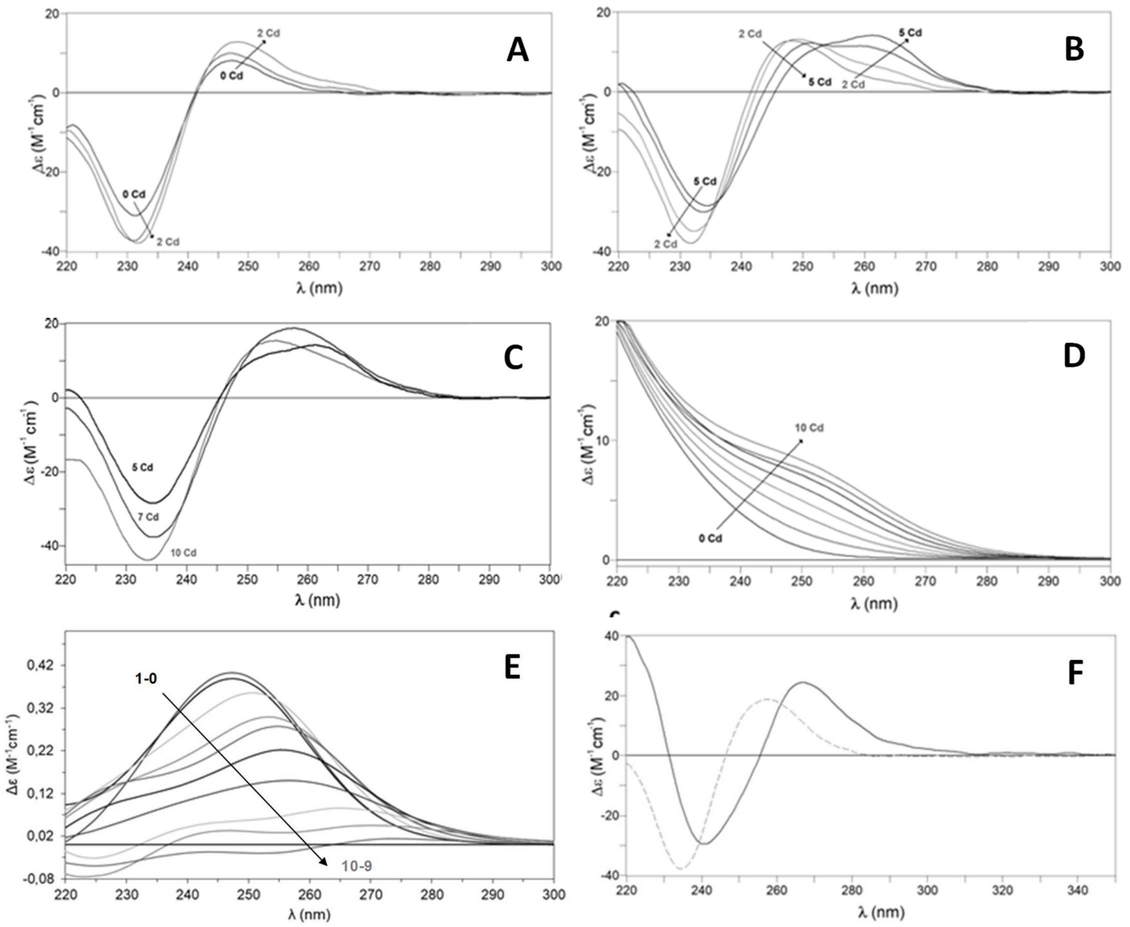


**Figure S3.** Spectroscopic characterization of the Zn/Cd replacement in the Zn-PbrMT2 preparation followed by (A, B and C) CD and (D) UV-vis. (E) UV-Vis difference spectra. (F) Comparison of CD spectra of *in vivo* Cd-PbrMT2 species (solid line) and the *in vitro* Cd-PbrMT2 species (dashed line) after adding 7 equivalents of Cd(II) to Zn-PbrMT2 production.

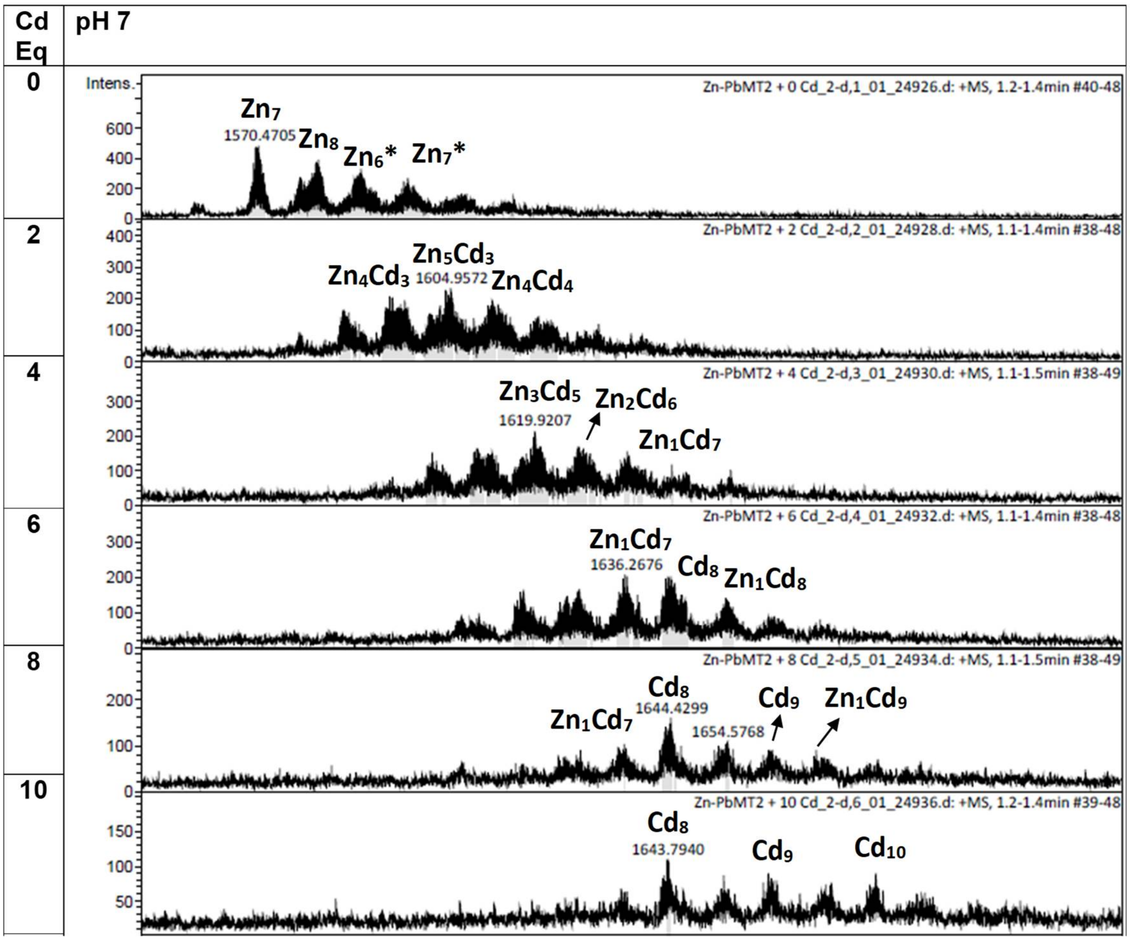


**Figure S4.** ESI-MS spectra of the Zn/Cd replacement experiment performed on the Zn-PbrMT2 production at pH 7.
